# Using decision fusion methods to improve outbreak detection in disease surveillance

**DOI:** 10.1186/s12911-019-0774-3

**Published:** 2019-03-05

**Authors:** Gaëtan Texier, Rodrigue S. Alldoji, Loty Diop, Jean-Baptiste Meynard, Liliane Pellegrin, Hervé Chaudet

**Affiliations:** 1 French Armed Forces Center for Epidemiology and Public Health (CESPA), SSA, Camp de Sainte Marthe, 13568 Marseille, France; 2 UMR VITROME, IRD, AP-HM, SSA, IHU-Méditerranée Infection, Aix Marseille Univ, 13005 Marseille, France; 3 CESP, Univ. Paris-Sud, UVSQ, INSERM, Université Paris-Saclay, Villejuif, France; 4 Cancer and Radiation Team, Gustave Roussy Cancer Center, F-94805 Villejuif, France; 5 International Food Policy Research Institute (IFPRI), Regional Office for West and Central Africa Regional Office, 24063 Dakar, Sénégal; 6 UMR 912 - SESSTIM - INSERM/IRD/Aix-Marseille Université, 13385 Marseille, France

**Keywords:** Decision support system, Disease surveillance system, Decision making, Decision fusion, Outbreak detection, Bayesian network

## Abstract

**Background:**

When outbreak detection algorithms (ODAs) are considered individually, the task of outbreak detection can be seen as a classification problem and the ODA as a sensor providing a binary decision (outbreak yes or no) for each day of surveillance. When they are considered jointly (in cases where several ODAs analyze the same surveillance signal), the outbreak detection problem should be treated as a decision fusion (DF) problem of multiple sensors.

**Methods:**

This study evaluated the benefit for a decisions support system of using DF methods (fusing multiple ODA decisions) compared to using a single method of outbreak detection. For each day, we merged the decisions of six ODAs using 5 DF methods (two voting methods, logistic regression, CART and Bayesian network - BN). Classical metrics of accuracy, prediction and timelines were used during the evaluation steps.

**Results:**

In our results, we observed the greatest gain (77%) in positive predictive value compared to the best ODA if we used DF methods with a learning step (BN, logistic regression, and CART).

**Conclusions:**

To identify disease outbreaks in systems using several ODAs to analyze surveillance data, we recommend using a DF method based on a Bayesian network. This method is at least equivalent to the best of the algorithms considered, regardless of the situation faced by the system. For those less familiar with this kind of technique, we propose that logistic regression be used when a training dataset is available.

**Electronic supplementary material:**

The online version of this article (10.1186/s12911-019-0774-3) contains supplementary material, which is available to authorized users.

## Background

The task of outbreak detection can be considered as a classification problem, and outbreak detection algorithms (ODAs) can be viewed as classifiers or sensors providing a binary decision (outbreak yes or no) for each time step of surveillance. For specialists in charge of a disease surveillance system, with more than 120 ODAs published [1] and in the absence of a consensus among specialists, the task of choosing the best ODA remains an highly complex one [2, 3]. Indeed ODA performance depends on several characteristics associated with the outbreak curve (shape, duration and size), the baseline (mean, variance) [4, 5] and their relationships (signal-to-noise ratio, signal-to-noise difference) [6, 7]. In this context, the hope of having a single algorithm that would be efficient enough to detect all outbreaks in all situations faced by a disease surveillance system is probably illusory.

For that reason, certain teams in charge of disease/syndromic surveillance systems choose to work with several ODAs to analyze the same surveillance dataset [8] as a multisensor system [9] with the objective of being able to produce correct decisions with a given amount of input information. Even if multiple sensors provide significantly more information on which to base a decision than a single sensor, using multiple classifiers or sensors can lead to several issues. Among them, as detailed in [9, 10], we can cite data conflict (agreement between classifier decisions), uncertainty, correlation, imprecision, incompleteness…, all of which makes decision fusion (DF) a challenging task. Finally, all these problems call into question the true benefit of using multiple ODAs for decision-making.

If we consider ODA decisions as a whole, the outbreak detection problem should be treated as a decision fusion problem of multiple classifiers/sensors. Decision fusion methods are tailored to generate a single decision, from multiple classifiers or biometric sensor decisions [11]. Fusion also provides the advantage of compensating for the deficiencies of one sensor by using one or more additional sensors. Moreover, in the context of surveillance, most of these techniques are automatable and can be added to the decision support system integrated in a disease surveillance system.

There are numerous publications on fusion methods for outbreak detection focused on the fusion of data collected from multiple streams [12–17] using different methods, such as Bayesian Networks, to manage different sources of data potentially useable in surveillance. However, to our knowledge, only one work [18] describes a decision fusion method applied to a single data stream. This study used an approach to enhance the classifier structure and yielded ambivalent results, according to the authors. The study’s limitations and the conceptual framework of Dietterich’s reasons (statistical, computational and representational) [19], justifying why multiple classifiers may work better than a single one, suggest the necessity of new studies in this field.

With the aim of improving decision making for disease surveillance system users, we propose to evaluate the benefit of using DF methods fusing multiple ODA decisions versus using a single method of outbreak detection.

This study is a proof of concept that aims at evaluating the capabilities of DF methods to enhance the reliability of outbreak detection systems. For this purpose, we will use synthetic data for controlling the outbreak curve characteristics in place of real data, which don’t allow the experimental controls required for this study.

## Methods

### Datasets

#### Evaluation

In the lack of a consensual gold standard allowing the delineation of a real outbreak within a disease surveillance series [7], the necessity to control precisely the onset and the end of the outbreak signal and finally to obtain a sufficient sample size to allow an adequate evaluation, we choose, as several authors (Buckeridge, Jackson..), to use synthetic data. A more complete discussion on this subject can be found in Texier and al [20].

The simulated data sets were generated according to approaches already detailed in previous studies [4, 7, 20]. Each simulated dataset was generated by combining two components: a baseline and outbreak signals. In this work, given a minimum outbreak spacing of 15 days between two outbreaks, the outbreak signals were randomly superimposed on baseline data in order to respect 10 ± 1% of the prevalence of outbreak days over 20 years. Five levels of baseline were generated, corresponding to the expected daily incidences of 1, 3, 5, 10 and 30 cases per day. Based on a real outbreak of Norovirus which had already been published [21], we used a resampling method [4, 7, 20], to generate curves with four different outbreak magnitudes (10, 30, 50 and 100 cases) and with a same duration of 12 days, corresponding to the duration of the originating real outbreak. Depending on the influence of the curve shape on ODA evaluation results, we considered that the use of resampling methods for generating our epidemic curves was the most realistic (see on this topic [20]). Twenty evaluation datasets (corresponding to the different combinations of the 5 levels of baseline with the 4 levels of outbreak magnitudes) were produced. We calculated the sample size required to estimate our evaluation metrics (as the sensitivity defined by Jafarpour [22]) with a specified level of confidence and precision (with a maximal error allowed of 3%). To reach this objective of precision, each algorithm had to evaluate 1100 outbreaks during this study. Finally, our evaluation datasets corresponded to 146,000 simulated days of surveillance that were evaluated by each sensor.

#### Training

For methods requiring a learning period, we simulated data with a 5-year surveillance period. Training and evaluation datasets were generated independently but had similar characteristics in terms of baseline level, outbreak size, and prevalence. We used exactly the same training dataset for all the learning methods.

### Outbreak detection algorithms

In this study, we used a set of six outbreak detection algorithms frequently used in routine disease surveillance systems [8], for which several statistical packages [23] are available and which are easily implementable. We chose the Cumulative Sum (CUSUM) chart as proposed by Rossi [24], the C-family of detection algorithms (C1, C2, and C3), which are adaptive algorithms included in the Early Aberration Reporting System (EARS) developed by the Centers for Disease Control and Prevention (CDC) [25], the Exponential Weighted Moving Average algorithms (EWMA) [6], and the Farrington algorithm, which should be applicable to various types of infections [26].

### Decision fusion methods (DFMs)

#### Taxonomy and choice

Fusion of data/information can be carried out on three levels of abstraction: data fusion, feature fusion, and classifier fusion (also referred to as decision fusion or mixture of experts) [27]. Due to the large number of classifier fusion methods in the literature, we decided to base our choice of methods on a taxonomy of these techniques proposed by Ruta [28]. Based on individual classifier outputs, Ruta identified two main approaches to combining classifiers, namely classifier selection (or structure optimization) and classifier fusion. The first approach looks for the single best classifier or a selected group of classifiers and uses only their outputs to build a final decision or for further processing.

The second approach focuses on classifier outputs and combines those outputs. According to the characteristics of the combined outputs, several authors have identified three levels of aggregation [28–30]:

- The measurement level: A classifier attributes a probability value to each label
- The rank level: A classifier ranks all labels in a queue and chooses the top label
- The abstract level (or single class label): A classifier only generates a single-label output (in our case, outbreak yes or no).

These three levels form an information gradient where the measurement level contains the most information and the abstract level contains the least [30].

We selected two simple and intuitive methods from the abstract level: the majority voting scheme and the weighted voting scheme.

The second level aims at reordering a class set. Logistic regression methods, which are situated at this level and are well known to epidemiologists, assign a weight to each classifier reflecting its importance in an efficient multiple sensor system. In this category, we also selected the CART Method [31].

The largest group of classifier fusion methods associated with the measurement level produces output values in the [0–1] range. These values cover all known measures of evidence (probability, possibility, necessity, belief, and plausibility) and are tailored to quantify a level of uncertainty. Indeed, all the fusion methods in this group try to reduce the level of uncertainty by maximizing a measure of evidence [28]. From this group, we selected the Bayesian Belief Networks method. A brief synopsis on each decision fusion method chosen is provided below.

#### Voting methods

The simplest way to combine the decisions of multiple outbreak detection algorithms is by voting, which corresponds to performing a linear combination of the prediction results of the algorithms. In the case of majority voting (MV) scheme fusion, the method gives equal weight to the decisions and carries out the prediction with the highest number of votes as the result. Weighted majority voting (WMV) stems from relaxing the assumption about equal individual accuracies. We choose area under the ROC Curve to weight the vote. Indeed, the AUC, which is based on both sensitivity and specificity, can be considered as a relevant indicator of algorithm performance to weight the vote, increasing the participation of decision with high sensitivity and specificity.

The reader is referred to Rahman et al. [32] for a comprehensive examination of the subject.

#### Logistic regression

The logistic regression model relates the conditional probability of an event distributed as a binomial Y according to a weighted combination of values for variables such as X_1_,X_2_,…,X_n_ which represent the decision of each outbreak detection algorithm (suppose j(1 ≤ j ≤ n) then X_j_ = 1 or X_j_ = 0) [33]. Y is the response variable corresponding to the true value for outbreak generated in the simulated data, while the various X’s, usually called explanatory variables, are ODAs. As for the weighted voting scheme, logistic regression can be seen as a linear combination (y = ß_1_ X_1_ + ß_2_ X_2_ + … + ß_i_ X_i_) of ODA decisions X_i_ weigthed by an estimated coefficient ß_i_. To estimate the model coefficients (ß_i_), the logistic regression was run on the training dataset. The selection of the final model in the training step was based on the lowest Akaike Information Criterion (AIC). In the end, the model selected was used on the simulated data having a 20-year surveillance period. On any given day, the results of the ODAs provide a predicted value of Y, representing the probability of an outbreak on that day. If this predicted probability exceeds 0.5, we classify the day as an outbreak day.

#### Classification and regression trees (CART)

CART is a classification method that has been successfully used in many health care applications [34]. Although there are variants of tree-based methods with different splitting criteria, CART was selected for this study, since it is used in decision fusion [35]. The reader is directed to Breiman [36] for a comprehensive description of the CART algorithm.

The six decisions of the ODA are used as independent variables in our CART model. As with logistic regression, the training data sets were used for the construction of maximum tree and for the choice of the right tree size. The *rpart* package of R software was used for the implementation of the CART model [37].

#### Bayesian networks (BNs)

Bayesian Networks (BNs) belong to the family of directed acyclic graph models. The network structure can be described as follows: Each node in the graph represents a binary variable provided by each classifier (i.e. ODA), while the edges between the nodes represent probabilistic dependencies among the corresponding variables.

As with the two previous DF methods (logistic regression and CART), the dataset generated during a 5-year surveillance period was used to train the BN. The *bnlearn* R package [38, 39] and Netica [40] were used to implement the BN. To validate the Bayesian network structure from our data, we used the Hill Climbing algorithm based on the Bayesian Information Criterion (BIC) score. An estimated probability of epidemic presence is provided by the BN and a probability threshold of 50% was selected to classify the outbreak presence/absence status for a given day, as in logistic regression.

### Evaluation metrics

We evaluated the performance metrics using several criteria: accuracy, prediction quality, and timeliness of outbreak detection. Accuracy was assessed by the specificity (Sp), the sensitivity (Se), and the area under the ROC (Receiver Operating Characteristic) curve (AUC) [22]. Two variants of Se were calculated in the paper: Se per day, which is the probability of correctly classifying outbreak days, and Se per outbreak, which is the ability to detect at least one outbreak day over the entire duration of the outbreak.

The evaluation of the quality of predictions was done using positive and negative predictive values (PPV and NPV respectively). The timeliness of outbreak detection was evaluated using the time to detection, the proportion of cases required for outbreak detection, the weighted AUC and the area under the Activity Monitor Operating Characteristic (AMOC) curve. The time to detection was defined as the mean and median number of days from the beginning of each outbreak to the first alarm during the outbreak. The proportion of cases required for outbreak detection was defined as the number of cases already occurring by the moment of detection divided by the total number of cases in the outbreak. This quantity can be seen as the minimal number of outbreak cases required for outbreak detection. The area under weighted ROC (AUWROC) is an ROC curve in which each point of the curve is weighted by a timeliness measure [41] and the area under the AMOC curve represents the relationship between the timeliness of outbreak detection and the false alarm rate (1-Specificity) [42]. A timeliness score defined as the proportion of time saved by detection relative to an outbreak onset, was also calculated as follows:

$$ \mathrm{Timeliness}\ \mathrm{score}=1-\frac{\mathrm{time}\ \mathrm{detection}-\mathrm{time}\ \mathrm{onset}\ }{\mathrm{Outbreak}\ \mathrm{duration}} $$

where outbreak duration is the total outbreak length in days, time detection is the index of the day within the time series when the outbreak is detected and time onset is the index of the day on which outbreak starts [22]. The timeliness score is 1 if the outbreak is detected on the first day of occurrence and 0 when the outbreak is not detected [6].

We also assessed the influence of the outbreak and baseline characteristics on the performance metrics of the ODAs and the DF methods. As defined in a previous study, the signal-to-noise difference (SND) was used for this evaluation [7]. In practice, three scenarios corresponding to three values of SND were considered: positive, quasi-null and negative SND. A positive SND corresponds to a higher number of cases in the outbreak than in the baseline during the outbreak period, and a negative SND to the opposite.

All algorithms, DF methods, and analyses were implemented with R software 3.3.0 [23] using the following packages: surveillance (for most algorithms), qcc (for EWMA), flux (for the estimations of AUCs), rpart and rpart.plot (for CART), bnlearn (Bayesian Networks).

## Results

### Accuracy and quality of prediction assessment

Table 1 summarizes the performance metrics of accuracy for six ODA and five DFM in terms of detection sensitivity per outbreak or per day, specificity, PPV, NPV and AUC. The six outbreak detection algorithms had a detection sensitivity per outbreak ranging from 72 to 89%, with the lowest for the C1 algorithm and the highest for the EWMA algorithm. The implementation of DFM showed that voting methods provided detection sensitivities per outbreak [78 to 82%], close to those of CUSUM, C3 or Farrington while other DFMs such as logistic regression, CART, or BN, had on average a detection sensitivity per outbreak lower than the range indicated above. The detection sensitivity per day varied strongly from 10 to 45% for the ODAs. This metric was more stable among the DFM, as it varied only from to 23 to 27%.

**Table 1:** Performance metrics for the accuracy and prediction quality of the outbreak detection algorithms and the decision fusion methods

	Sensitivity per outbreak		Sensitivity per day		Specificity		PPV		NPV		AUC	
	Mean	STD	Mean	STD	Mean	STD	Mean	STD	Mean	STD	Mean	STD
CUSUM	0.83	0.28	0.45	0.29	0.87	0.17	0.49	0.35	0.94	0.03	0.73	0.14
C1	0.72	0.34	0.10	0.07	0.99	0.00	0.38	0.21	0.92	0.01	0.53	0.02
C2	0.74	0.33	0.16	0.11	0.99	0.00	0.45	0.23	0.92	0.01	0.57	0.04
C3	0.82	0.25	0.25	0.14	0.96	0.00	0.36	0.16	0.93	0.01	0.62	0.07
Farrington	0.86	0.20	0.20	0.11	0.97	0.02	0.51	0.33	0.92	0.01	0.66	0.10
EWMA	0.89	0.20	0.29	0.17	0.95	0.02	0.37	0.20	0.93	0.02	0.64	0.09
Majority voting	0.82	0.26	0.24	0.17	0.99	0.01	0.61	0.32	0.93	0.02	0.60	0.09
Weighted majority voting	0.78	0.31	0.23	0.17	0.99	0.01	0.66	0.33	0.93	0.02	0.61	0.08
Logistic regression	0.65	0.44	0.27	0.25	1.00	0.00	0.90	0.06	0.93	0.02	0.70	0.12
CART^a^	0.65	0.44	0.26	0.24	1.00	0.00	0.91	0.07	0.93	0.02	0.69	0.12
Bayesian Networks	0.66	0.43	0.26	0.24	1.00	0.00	0.90	0.09	0.93	0.02	0.70	0.12

^a^*CART* Classification and Regression Trees, *PPV* Positive Predictive Values, *NPV* Negative Predictive Values, *AUC* Area Under the ROC (Receiver Operating Characteristic) Curve, *STD* Standard Deviation

Concerning the quality of outbreak prediction, PPVs were ranged from 36 to 51% for the outbreak detection algorithms, and was higher for the five DFMs starting at 61% and reaching more than 90% for the three DFMs using a learning step (logistic regression, CART, or Bayesian networks). Thus, when the best algorithm had one chance in two to correctly predict the outbreak status for a given day, the best fusion methods had nine out of ten chances not to be mistaken. However, NPVs were almost identical between the outbreak detection algorithms and the fusion methods.

**Fig. 1:**
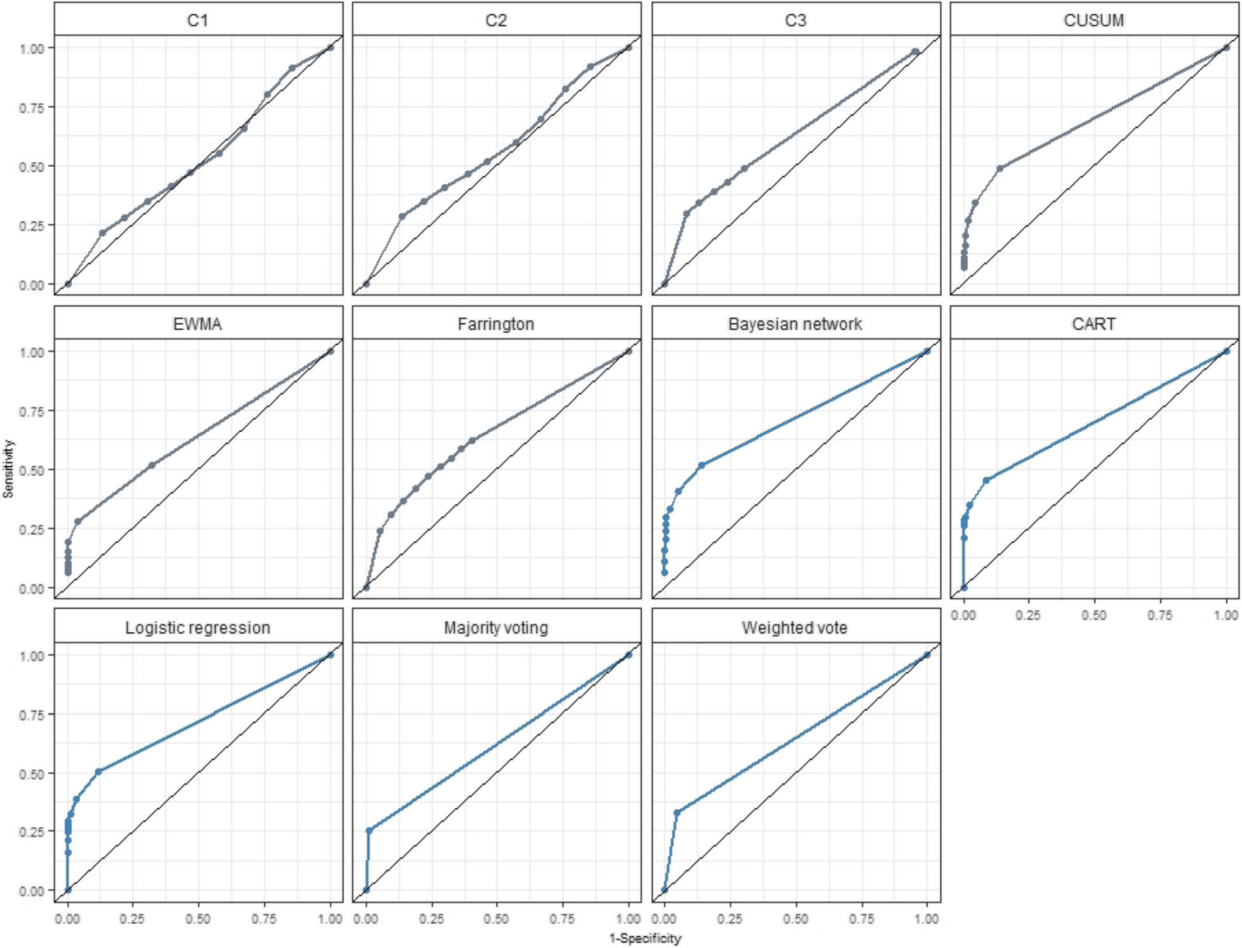
Accuracy measured by area under curve (AUC) according to outbreak detection algorithm and decision fusion method

Our evaluation results show that the three DFMs using a learning step yielded overall accuracies that were quite close to that found for CUSUM, which consistently provided the highest accuracy (AUC =73%) among outbreak detection algorithms (see Fig. 1).

### Timeliness assessment

Timeliness is a key metric for early warning surveillance systems. It refers to the ability of the detection algorithm to detect a signal aberration early enough to enable public health authorities to respond rapidly. Among the outbreak detection algorithms, the best timeliness was achieved by the EWMA algorithm (cases required = 41%, time to detection = 5.28, proportion of delay = 38%) [Table 2]. For the DFMs, the simplest react the most rapidly. In general, fusion methods were slightly slower than detection algorithms. But when we weighted timeliness by integrating accuracy metrics to reflect the fact that a rapid false alarm is of relatively little value, DFMs produced similar results, in terms of AMOC or AUWROC, to that provided by the CUSUM algorithm, which was the fastest detection algorithm.

**Table 2:** Performance metrics for the timeliness of outbreak detection of the detection algorithms and decision fusion methods

	Cases required		Proportion of delay		Time to detection		AMOC		AUWROC	
	Mean	STD	Mean	STD	Mean	STD	Mean	STD	Mean	STD
CUSUM	0.47	0.24	0.41	0.20	5.10	2.83	0.83	0.05	0.66	0.11
C1	0.54	0.27	0.49	0.23	6.50	3.44	0.87	0.03	0.50	0.03
C2	0.52	0.27	0.48	0.23	5.90	3.09	0.86	0.03	0.54	0.05
C3	0.56	0.18	0.46	0.16	6.30	2.64	0.82	0.04	0.56	0.07
Farrington	0.46	0.17	0.41	0.14	5.23	2.26	0.87	0.04	0.61	0.10
EWMA	0.41	0.19	0.38	0.14	5.28	2.45	0.87	0.03	0.59	0.08
Majority voting	0.49	0.22	0.44	0.18	5.30	2.56	0.75	0.11	0.57	0.07
Weighted majority voting	0.53	0.24	0.47	0.20	5.43	2.54	0.75	0.11	0.57	0.07
Logistic regression	0.59	0.31	0.56	0.30	7.15	3.82	0.82	0.07	0.63	0.10
CART^a^	0.60	0.30	0.57	0.30	7.10	3.85	0.77	0.12	0.62	0.09
Bayesian networks	0.60	0.30	0.56	0.29	6.75	3.55	0.81	0.09	0.63	0.11

^a^*CART* Classification and Regression Trees, *Cases required* proportion of cases needed for outbreak detection, Proportion of delay = 1 – timeliness score, that is: 1- (sum of time to detection) / outbreak duration, *AMOC* Activity Monitor Operating Characteristic, *AUWROC* Area Under Weighted ROC, *STD* Standard Deviation

### The influence of signal-to-noise difference on outbreak detection performance

From our results, it is clear that the SND has a direct impact on the timeliness and the capacity of outbreak detection, whatever method was used. Firstly, when the outbreak signal is easy to detect among the baseline noise, the best performance in terms of detection is provided by the Farrington algorithm (Specificity = 100%, PPV = 99%, NPV = 95%, AUWROC = 79%) [Table 3]. Overall, fusion methods seem to perform at the same level as the best ODA when SND is positive. It should be noted that when the SND tends towards zero, fusion methods even seem to provide a slight improvement over ODAs. Then, when the outbreak signal is more difficult to detect among the baseline noise, the best performance in terms of detection is provided by the CUSUM algorithm (PPV = 96%, NPV = 91%, AUWROC = 59%) but when timeliness is considered more important than PPV, EWMA (time to detection = 5, proportion of delay = 37%, AUWROC = 54%) and the Farrington algorithm (time to detection = 5, proportion of delay = 55%, AUWROC = 56%), can be considered as a good compromise that comes at the price of a high rate of false alarms when the SND is negative (PPV = 25 to 46%).

**Table 3:** Influence of signal-to-noise difference (SND) characteristics on the performance metrics of the detection algorithms and the fusion methods

	Sensitivity per outbreak	Sensitivity per day	Specificity	PPV	NPV	Cases required	Proportion of delay	Time to detection	AUC	AMOC	AUWROC
Positive SND: scenario with a SND = 65.4											
CUSUM	1	0.74	0.83	0.29	0.97	0.17	0.25	4	0.89	0.90	0.81
C1	1	0.25	0.99	0.69	0.93	0.08	0.15	2	0.59	0.92	0.57
C2	1	0.38	0.99	0.77	0.94	0.08	0.14	2	0.66	0.92	0.64
C3	1	0.54	0.96	0.61	0.95	0.09	0.19	2	0.77	0.90	0.72
Farrington	1	0.42	1.00	0.99	0.95	0.18	0.21	2	0.84	0.93	0.79
EWMA	1	0.58	0.97	0.67	0.96	0.14	0.22	2	0.76	0.90	0.70
Majority voting	1	0.56	1.00	0.98	0.96	0.10	0.17	2	0.78	0.91	0.73
Weighted majority voting	1	0.53	1.00	0.99	0.96	0.13	0.22	2	0.77	0.89	0.71
Logistic regression	1	0.59	0.99	0.92	0.96	0.09	0.16	2	0.84	0.94	0.80
CART^a^	1	0.56	1.00	0.99	0.96	0.12	0.19	2	0.83	0.92	0.78
Bayesian Networks	1	0.56	1.00	1.00	0.96	0.12	0.19	2	0.90	0.93	0.84
Quasi-null SND: scenario with a SND = −1.4											
CUSUM	1	0.61	1.00	0.93	0.96	0.49	0.38	5	0.86	0.88	0.77
C1	1	0.17	0.99	0.64	0.92	0.24	0.27	4	0.55	0.89	0.53
C2	1	0.28	0.99	0.75	0.93	0.24	0.26	4	0.61	0.89	0.58
C3	1	0.39	0.97	0.56	0.94	0.36	0.31	5	0.72	0.86	0.66
Farrington	1	0.27	1.00	1.00	0.93	0.35	0.34	4	0.80	0.91	0.74
EWMA	1	0.51	0.94	0.46	0.95	0.20	0.24	4	0.76	0.90	0.70
Majority voting	1	0.42	1.00	0.99	0.94	0.25	0.28	4	0.71	0.86	0.65
Weighted majority voting	1	0.38	1.00	1.00	0.94	0.34	0.33	4	0.50	0.50	0.50
Logistic regression	1	0.70	0.99	0.93	0.97	0.22	0.27	4	0.86	0.88	0.77
CART^a^	1	0.68	1.00	0.93	0.97	0.25	0.27	4	0.84	0.86	0.75
Bayesian Networks	1	0.70	0.99	0.94	0.97	0.23	0.27	4	0.86	0.88	0.77
Negative SND: scenario with a SND = −89.2											
CUSUM	0.29	0.03	1.00	0.96	0.91	0.87	0.77	11	0.65	0.82	0.59
C1	0.51	0.05	0.99	0.25	0.91	0.73	0.64	5	0.52	0.86	0.49
C2	0.60	0.07	0.98	0.30	0.91	0.70	0.60	5	0.55	0.86	0.51
C3	0.78	0.16	0.96	0.27	0.92	0.62	0.50	6	0.59	0.82	0.54
Farrington	0.67	0.09	0.99	0.46	0.92	0.64	0.55	5	0.60	0.87	0.56
EWMA	0.98	0.18	0.95	0.25	0.92	0.47	0.37	5	0.59	0.87	0.54
Majority voting	0.60	0.07	0.99	0.45	0.92	0.71	0.61	5	0.53	0.69	0.51
Weighted majority voting	0.53	0.06	1.00	0.69	0.91	0.75	0.65	5	0.55	0.72	0.52
Logistic regression	0.29	0.03	1.00	0.96	0.91	0.87	0.77	11	0.60	0.81	0.55
CART^a^	0.29	0.03	1.00	0.96	0.91	0.87	0.77	11	0.59	0.77	0.54
Bayesian Networks	0.51	0.06	1.00	0.96	0.91	0.80	0.68	7	0.60	0.81	0.55

^a^*CART* Classification and Regression Trees, *PPV* Positive Predictive Values, *NPV* Negative Predictive Values, *AUC* Area Under the ROC (Receiver Operating Characteristic) Curve, *Cases required* proportion of cases needed for outbreak detection, Proportion of delay = 1 – timeliness score, that is: 1- (sum of time to detection) / outbreak duration, *AMOC* Activity Monitor Operating Characteristic, *AUWROC* Area Under Weighted ROC. Positive SND: scenario generated with a daily incidence of 1 for the baseline and an outbreak magnitude of 100 (SND = 65.4), *Quasi-null SND* scenario generated with an daily incidence of 1 for the baseline and an outbreak magnitude of 30 (SND = −1.4), *Negative SND* scenario generated with a daily incidence of 3 for the baseline and an outbreak magnitude of 10 (SND = −8)

## Discussion

### Evaluation of decision fusion

#### Majority voting

The voting method is the simplest DF method to implement, since it doesn’t require a priori knowledge. Whatever the situation, to guarantee the best results for the voting method, it is better to use an odd number of independent ODAs [43]. The main qualities of this method are probably its timeliness (with only 49% of total number of outbreak cases required on average before a detection and a proportion of delay = 0.44) with a detection occurring on average 5.3 days after the onset of the outbreak, with relatively good performance as long as the SND remains positive. Another advantage is its simplicity of implementation and the possibility of changing the decision rule with the aim of optimizing detection. Here, we chose a majority voting decision rule, but others exist, such as Byzantine, unanimity, or m-out-of n voting rules [44].

Theoretically promising compared to the above technique (by overweighting the most efficient ODA), Weighted majority voting ultimately suffers from the limitations of voting methods without the advantage in terms of reactivity offered by the simple voting method. Xu [29], and several authors have compared this approach to other DF methods and find that this method usually underperforms, as it did in our study.

#### Logistic regression

In the logistic regression method, the logit provides an estimated probability of an outbreak. In our experiment, we used the theoretical optimal threshold of 0.5 as the decision rule, as suggested by Verlinde [45], to confirm or invalidate the alarm. But, decision threshold fixed at 0.5 should be adjusted to improve sensitivity, specificity and predictive value by using another experimentally-determined threshold [46].

As explained in Verlinde, one advantage of logistic regression is the possibility to consider ß_i_ parameters as direct measure of the relative importance of an ODA. It minimized the total error rate, (combining with the same weight, false alarm rate and false negative rate) with a low rate of false alarms (0.0%) compared with decision tree (0.3%) and majority voting (3.2%), but a higher rate of false negative days (2.7%) compared with decision tree (7.7%) and majority voting (0.0%). Verlinde, Altmann also considered logistic regression to be the best meta-classifier [47] according to the AUC and accuracy criteria. According to these authors, logistic regression is useful when the different experts show significant differences in terms of accuracy and is also considered a robust method.

#### Cart

Like logistic regression, CART can be used for ODA selection and ranking by identifying the most important sensors (near the root node). Because CART makes no assumption about the underlying distribution, this point can be considered an advantage, in comparison with logistic regression models, particularly when the data are far from the (multivariate) normal distribution [34].

However, we agree with several authors in finding that tree structure learned from data is very sensitive to a small change in the training data set and provides very different splits, ultimately making interpretation somewhat precarious [35, 48]. And according to the type of dataset, a change in the split criteria can lead to the creation of very different trees. In addition, the different threshold parameters of the rpart algorithm did not allow us to improve prediction performance, especially in the datasets with a very low SND. According to the literature, the major reason for this instability is the hierarchical nature of the process: The effect of an error in the top split is propagated down to all of the splits below it. The performance of CART was consistently good, but slightly below that of the regression models and BN, and was always more accurate than voting scheme methods. The difficulty in identifying the right settings remains a problem.

#### Bayesian network

Our evaluation results show that, whatever the outbreaks and baseline characteristics, logistic regression and the Bayesian Networks were able to achieve detection with high accuracy (AUC = 0.70 – Table 1), which is similar to the best algorithm performance (AUC = 0.73). The ROC curve comparison for the prediction of “detection” presented in Fig. 1 shows that DFM with a training step performs as well as the best ODA (CUSUM: AUC = 0.73).

Considering that an NPV around 0.93 was found for all methods (ODA and DFM), we observed a major gain (77%) in terms of positive predictive values (PPV) by using DFMs (BN, logistic regression and CART methods: PPV around 90%) compared to the best ODA (Farrington: PPV = 51%), which also requires a 5-year training period.

Bayesian methods are less reliant on specific asymptotic results, a property that can be a hindrance when employing frequentist methods in small sample contexts [49]. Another advantage of a Bayesian model is that there is no a priori hypothesis about the nature of the modeled relationships [50]. Like other DF “learning” methods we noticed that, occasionally, BN depends on the learning step, making this method sensitive to that step. Another advantage of BN models is their capacity to enrich their “surveillance knowledge” from new cases to update their probability tables even if the surveillance practices may change over time. This continuous training [47] enables the model to be updated and its predictive quality to be improved, allowing outbreak detection to be tailored to each surveillance system.

### Using decision fusion for real time detection

Provided that the BN graph was adapted to the surveillance dataset, tools like NETICA© make it possible to visualize and calculate the conditional probability associated with each real-time ODA decision (Additional file 1: Table S1). Unlike other decision fusion methods, this dynamic tool also makes it possible to take into account the order in which results appear. For example, during the structured learning step of our experiment with our dataset based on a baseline at 1 and a signal at 30 for a real outbreak day, we identified three algorithms of interest: CUSUM, EWMA, and C3. We observed that when the CUSUM ODA triggers an alarm alone, while all the other ODAs remain silent, the probability of an outbreak is estimated at 81.0%. It grows to 96.8% if the second alarm is produced by EWMA and to 98.7% if the third is produced by C3. Results are modified as follows if the alarm sequence is EWMA/CUSUM/C3: 5.4, 96.8, 98.7%. However, if we take into account a new alarm (the fourth) triggered by an ODA with a non-significant link to the outbreak status, for example in this case the C1 algorithm, the probability falls to 50%, showing the importance of the training period for methods for which contributing ODAs need to be selected.

We agree with Jafarpour [22] that inference performed using a BN can help to develop what-if analyses in disease surveillance activity or to identify an efficient ODA configuration and combination given the desired level of detection performance. This type of tool provides insight into the features of detection methods that are important to optimize to obtain better detection.

### Decision fusion: benefits and limitations

In this study, we try to quantify the value of decision fusion (proof-of concept) in disease surveillance by using a simulated dataset standardized (allowing reproducible evaluation). The choice to use 20 years’ period was only driven by sample size constraints required for statistical precision in our study. This level of background information would not be required for routine implementation. This period is an extreme situation because in the real life of surveillance, measurements and ecology of diseases are not consistent over the 20 years.

A number of extensions to this work may also improve the generalization of our study. First, we suggest before implementation to consider other kinds of outbreak curves in addition to our Norovirus outbreak. However, we have known since Buckeridge and Jackson [4, 5] that ODA performance results are influenced by curve shape. Our results were also affected by the quality of the training period for models requiring that step. In the absence of historical data or a realistic (for the population under surveillance) simulated dataset, we need to clarify and compare more precisely the use of a single ODA versus a decision fusion tool. That is why, before putting them into routine use, we advise epidemiologists to validate their decision fusion models in their own context of use, with their own data and especially by testing the different diseases habitually faced by their system.

As expected [7], the most informative determinants of detection performance was SND, which is a parameter combining the baseline levels and the peak size of the outbreak. However, one limitation in comparing surveillance and DF methods is the difficulty in choosing the evaluation metric to optimize. Indeed, and according to the aim and context of surveillance, people in charge of surveillance systems need to optimize either the PPV, the NPV, the timeliness, or a mix of these metrics (AUWROC, AMOC, etc.). This limitation was addressed in our work by proposing different evaluation metrics and surveillance circumstances (surveillance scenario).

Our results are a contribution to the fact that decision fusion models can decrease the risk of using a single inappropriate ODA. Indeed, this approach does not require the prior choice of an ODA, which could be unsuitable for a specific context. In this sense, choosing to use decision fusion is a way to control the risk of ODA misspecification and limitation. In most cases, a decision fusion model outperforms a single algorithm. These results support the conceptual framework of Dietterich’s reasons (statistical, computational, and representational) [19], that justify why multiple classifiers may work better than a single one.

Use of synthetic data in this work is only driven by our focus on reproducible assessments of performance across the different DF approaches. An in-depth application to real surveillance data is beyond the aim of this paper. But before any deployment of decision methods, in a real disease surveillance system using several algorithms on the same data, a confirmation step should be considered.

This work can be extended by including more fusion decision methods such as Dempster-Shaffer, fuzzy logic, Neural Network [28] /Deep Learning or by using the framework of decision spaces [51].

## Conclusions

Finally, our paper illustrates the fact that a good decision fusion method (as BN, logistic regression, or CART) is in our experiment at least equivalent to the best algorithm in terms of compromise between an early warning and the probability that the alarm triggered is a false alarm, whatever the situation being faced by the system, without the drawback of betting on the future. So, we recommend a decision fusion model based on a Bayesian Network approach to identify disease outbreaks in systems using several ODAs to analyze surveillance data. This conclusion doesn’t take into consideration other characteristics of surveillance system especially it’s stability, it’s human involvement and it’s resulting timeliness.

Numerous tools in the field of Bayesian Networks offer as an output a probability of outbreak presence/absence, thus making it possible to evaluate and readjust the decision threshold and real-time forecast. For those less familiar with this kind of technique, we suggest using logistic regression when a learning dataset is available. Otherwise, with a positive SND, a voting scheme technique can be considered in this specific circumstance.

In the future and once their parameters have been set, these statistical techniques could be integrated in decision support systems which will aim at providing assistance to expert decision making strategies during daily outbreak surveillance activities [52]. The major issues and challenges of such tools and techniques will be their adequacy to decision-related activities of these experts in outbreak context, described as real-setting, time-constrained, complex and uncertain situations [53, 54].

## Additional file

Additional file 1: **Table S1.** An example of 25 years of dataset (training dataset the first 5 years + evaluation dataset the next 20 years) used in this study to evaluate outbreak detection algorithm and decision fusion methods (Baseline = 3 cases by days in average, Total number of outbreak cases injected =50 cases). The baseline (Column A) level of disease surveillance corresponding to an average of 3 cases declared by days in the system and the complete outbreak signal corresponding to a total of 50 cases according a shape of Norovirus outbreak injected (Column B) several time in the baseline. Column C represents the first day of the outbreak (1 = Start of the outbreak) and Column D all days considered as epidemic (=1). (XLSX 195 kb)

## Abbreviations

DFM: Decision fusion methods
NPV: Negative predictive value
ODA: Outbreak detection algorithm
PPV: Positive predictive value
Se: Sensitivity
SND: Signal-to-noise difference
Sp: Specificity
