# Correction to: Using decision fusion methods to improve outbreak detection in disease surveillance

**DOI:** 10.1186/s12911-019-0802-3

**Published:** 2019-03-28

**Authors:** Gaëtan Texier, Rodrigue S. Allodji, Loty Diop, Jean-Baptiste Meynard, Liliane Pellegrin, Hervé Chaudet

**Affiliations:** 1 French Armed Forces Center for Epidemiology and Public Health (CESPA), SSA, Camp de Sainte Marthe, 13568 Marseille, France; 2 UMR VITROME, IRD, AP-HM, SSA, IHU-Méditerranée Infection, Aix Marseille University, 13005 Marseille, France; 3 CESP, Univ. Paris-Sud, UVSQ, INSERM, Université Paris-Saclay, Villejuif, France; 4 Cancer and Radiation Team, Gustave Roussy Cancer Center, F-94805 Villejuif, France; 5 International Food Policy Research Institute (IFPRI), Regional Office for West and Central Africa Regional Office, Dakar, 24063 Sénégal; 6 UMR 912 - SESSTIM - INSERM/IRD/Aix-Marseille Université, 13385 Marseille, France

**Correction to: BMC Med Inform Decis Mak**

**https://doi.org/10.1186/s12911-019-0774-3**

Following publication of the original article [1], the authors reported that one of the authors’ names is spelled incorrectly. In this Correction the incorrect and correct author name are shown. The original publication of this article has been corrected.

Originally the author name was published as:

- Rodrigue S. Alldoji

The correct author name is:

- Rodrigue S. Allodji
